# The images of psychiatry scale: development, factor structure, and reliability

**DOI:** 10.1186/s12888-014-0337-1

**Published:** 2014-12-11

**Authors:** Heather Stuart, Norman Sartorius, Tiina Liinamaa, The Images Study Group

**Affiliations:** Department of Public Health Sciences, Queen’s University, Kingston, Ontario, Canada; Association for the Improvement of Mental Health Programs, Geneva, Switzerland; Centre for Health Services and Policy Research, Queen’s University, Kingston, Ontario, Canada

**Keywords:** Stigma, Medical educators, Images of psychiatry scale, Efficacy of psychiatry scale

## Abstract

**Background:**

This analysis is based on a survey questionnaire designed to describe medical educators’ views of psychiatry and psychiatrists. Our goals in this paper were to assess the psychometric properties of the survey questions by (a) using exploratory factor analysis to identify the basic factor structure underlying 37 survey items; (b) testing the resulting factor structure using confirmatory factor analysis; and (c) assessing the internal reliability of each identified factor. To our knowledge, this is the first attempt to use these techniques to psychometrically assess a scale measuring the strength of stigma that medical educators attached to psychiatry.

**Methods:**

Survey data were collected from a random sample of 1,059 teaching faculty in 23 academic teaching sites in 15 countries. We conducted exploratory and confirmatory factor analysis to identify the scale structure and Cronbach’s alpha to assess internal consistency of the resulting scales.

**Results:**

Results showed that a two-factor solution was the best fit for the data. Following exploratory factor analysis, we conducted confirmatory factor analysis on a split half of the sample. Results highlighted several items with low loadings. Excluding factors with low correlations and allowing for several correlated variances resulted in a good fitting model explaining 95% of the variance in the data.

**Conclusions:**

We identified two unidimensional scales. The Images Scale contained 11 items measuring stereotypic content concerning psychiatry and psychiatrists. The Efficacy of Psychiatry Scale contained 5 items addressing perceptions of the challenges and effectiveness of psychiatry as a discipline.

## Background

Psychiatry has a problem with its image such that difficulty recruiting medical students into psychiatry has emerged as an important problem [1], being referred to by some as a “Sisyphean task” [2]. Studies carried out in different parts of the world—both developing and developed countries—indicate that medical students do not consider psychiatry as a desirable career choice [3-10]. In a survey of 655 students from 6 Australian medical schools, for example, psychiatry was identified as the least respected specialty and the least likely to be identified as a career choice [3]. In the Kingdom of Bahrain, only four of 140 (2.9%) medical students who completed the survey questionnaire from the Arabian Gulf University selected psychiatry as their first choice—the lowest of any specialty [9]. Similar proportions have been identified in the United States and the United Kingdom [11,12]. In a large 20-country study, 4.5% of the medical students surveyed indicated they were likely to choose psychiatry as a career [10].

Most countries have a shortage of psychiatrists and many rely heavily on international graduates [1,13]. In New Zealand, for example, the ratio of psychiatrists to population is 1 to 14,880; considerably lower than the often-cited benchmark of 1:10,000 [14]. In 53% of countries reporting to the World Health Organization, covering 69% of the world’s population, there is less than one psychiatrist per 100,000 population. This includes all countries in the South-East Asia Region and 96% of those in the African Region in which there is often no more than one psychiatrist per million population [15].

The bulk of research in this area has focused on the attitudes of medical students. Criticisms made by medical students are that psychiatry is too narrow in scope; it does not draw on all aspects of medical training; it is ineffective, unscientific and too emotionally demanding; and psychiatrists are unattractive role models. These negative attitudes persist even after contact with psychiatric educators and clinical rotations [7,16-18].

Research has demonstrated that a proportion of medical students will change their career choices due to negative comments (“badmouthing”) from mentors and peers. In a survey of 1,114 senior students, three quarters had heard some badmouthing about their career choice and 17% indicated that they had decided against their initial career choice because of negative comments that they heard about it. In over half of the cases, badmouthing was identified as coming from teaching faculty [19]. This research suggests that the attitudes of medical educators may be an important and understudied source of stigma against psychiatry as a career choice.

A review of the literature indicated that there were no psychometrically validated scales to measure medical educators attitudes. Therefore, we developed a pool of 37 Likert-type survey questionnaire items to describe medical educators’ views of psychiatry and psychiatrists. (We originally had 39 items but two items pertaining to psychiatric rotations were eliminated, as rotations were not offered in all of the countries participating in the study.) Given the lack of psychometrically tested instruments measuring attitudes of teaching faculty, and the importance of understanding medical educators views as a potential barrier to recruitment of psychiatrists, our goals in this paper were to: (a) identify the basic factor structure underlying the 37 items using an exploratory factor analysis; (b) test the resulting factor structure using a confirmatory factor analysis; and (c) assess the internal reliability of each identified scale. To our knowledge, this is the first attempt to psychometrically assess a scale to assess medical educators’ attitudes toward psychiatry and psychiatrists.

## Methods

### Study setting

This study was conducted as part of the scientific activities of the World Psychiatric Association’s Stigma and Mental Health Scientific Section. It was developed with the participants attending an international course on leadership skills for young psychiatrists in Asian/Pacific countries organized by the Association for the Improvement of Mental Health Programs, a not-for-profit organization in Geneva. Individuals attending the course agreed to participate in a research study in order to develop and improve their research skills. Subsequently several young psychiatrists from Europe petitioned to join the research group. Each participant functioned as the lead site investigator and was responsible for obtaining appropriate institutional approvals, conducting or coordinating the translation and back translation of the survey, and coordinating data collection.

### Sampling plan

Data were collected from 23 academic teaching sites in 15 countries: Belarus, China, Croatia, India, Indonesia, Iran, Japan, Portugal, Romania, Russia, Scotland, Singapore, Thailand, Turkey, and the Ukraine. The average response rate was 65% (1060 of 1629), with site-specific response rates ranging from 42% to 100% and sample sizes ranging from 25 to 169.

In each participating site, all non-psychiatric medical educators were enumerated from staff directories and their career stage (early, middle, or late) was determined. Individuals were considered to be in the early stage of their career if they were Lecturers, Assistant Professors, under ten years in practice, or under 40 years old. They were defined as in mid career if they were Associate Professors, had been in practice 11-25 years, or were 41-55 years old. Individuals were considered to be in late career if they were Full Professors, Emeritus Professors, in practice more than 25 years, or over the age of 56 years. A stratified random sample of 10 faculty members from each career stage (representing approximately 20% of teaching faculty overall) was then drawn from each site. Some sites over-sampled to support more detailed country-specific analyses. More detailed information on the methods and descriptive results can be found elsewhere [20].

### Item development

Our work was informed by three survey instruments, all of which measured attitudes of medical students: The ATP 30 scale developed by Burra and colleagues in 1982 [21], the 39-item questionnaire developed by Balon and colleagues in 1999 [22], and its predecessor, a 22-item questionnaire originally developed by Nielsen and Eaton in 1981 [23]. Only the ATP 30 had been psychometrically tested. Split-half reliability was high (0.9) and the six-week test-retest reliability in a control sample of first year medical students was 0.87.

Table 1 summarizes the genesis of our scale items. The first column shows the items that were obtained from the literature, with bolded references indicating the citation from which the item was drawn. In some cases, there were multiple similar items for a single idea. In others, there were gaps, which we filled with a new item. The second column shows the 37 revised scale items that were eventually tested and their corresponding survey item number. Five items were drawn from a single survey and used verbatim. Twenty-six items were adapted, and 6 items were added. Reworded and new items were reviewed by two of the authors (HS and NS). Items pertained to perceptions of psychiatry as a discipline (5); perceptions of the effectiveness of psychiatric treatments (7); perceptions of psychiatrists as role models (5); perceptions of psychiatry as a career (7); perceptions of psychiatric patients (7); and perceptions about the quality of psychiatric training (6).

**Table 1:** **Genesis of scale items**

**Original item(s)**	**Revised items for analysis and item number (total items for each construct)**
**Perceptions of psychiatry as a discipline (n = 5)**	
Psychiatry is unscientific and imprecise [22,23].	**Item 1:** Psychiatry is unscientific.
Psychiatry has very little scientific information to go on [21].	
Psychiatry is so unscientific that even psychiatrists can’t agree as to what its basic applied sciences are [21].	
Psychiatry is a rapidly expanding frontier of medicine [22,23]	**Item 2:** Psychiatry is a rapidly expanding frontier of medicine.
Psychiatry is attractive as a discipline because it is more intellectually comprehensive than other medical careers; it involves many fields of study including biology, psychology, sociology, history, philosophy and literature [23].	**Item 3:** Psychiatry is intellectually challenging.
Psychiatry is not a genuine and valid branch of medicine [22].	**Item 4:** Psychiatry is not a genuine and valid branch of medicine.
On average, psychiatrists make as much money as most other doctors [22].	**Item 5:** On average, psychiatrists make less money than other specialists.
On average, psychiatrists make less money than other physicians [23].	
**Perceptions of psychiatric treatments (n = 7)**	
Psychiatric research has made good strides in advancing care of the major mental disorders [22].	**Item 6:** Psychiatric treatments are not evidence based.
In recent years psychiatric treatment has become quite effective [21].	**Item 7:** Psychiatric treatments are as effective as treatments in other branches of medicine.
Psychiatric hospitals have a specific contribution to make to the treatment of the mentally ill [21].	**Item 8:** Psychiatric patients should be treated in specialized facilities.
Psychiatric treatment is helpful to most people who receive it [22].	**Item 9:** Most people who receive psychiatric treatment find it helpful.
With the forms of therapy now at hand most psychiatric patients improve [21].	
There is very little that psychiatrics can do for their patients [21].	**Item 10:** There is very little psychiatrists can do for their patients.
Psychiatric hospitals are little more than prisons [21].	**Item 11:** Psychiatric hospitals are little more than prisons.
Psychiatrists frequently abuse their legal power to hospitalize patients against their will [22,23].	**Item 12:** Psychiatrists have too much power over their patients.
**Perceptions of psychiatrists as role models (n = 5)**	
Most psychiatrists are clear, logical thinkers [22].	**Item 13:** Most psychiatrists are not clear, logical thinkers.
Psychiatrists are fuzzy thinkers [23].	
Attending psychiatrists during my psychiatry rotation were good role models [22].	**Item 14:** Psychiatrists are not good role models for medical students.
Psychiatrists understand and communicate with people better than the average physician [23].	**Item 15:** Psychiatrists are difficult to talk to.
Psychiatry is too analytical, theoretical, and psychodynamic, and not attentive enough to patients’ physiology [22].	**Item 16:** Psychiatrists are not attentive enough to physiology.
Psychiatry is a discipline filled with international medical graduates whose skills are of low quality [22].	**Item 17:** Psychiatry is filled with people whose medical skills are of low quality.
**Perceptions of psychiatry as a career (n = 7)**	
Friends and fellow students discouraged me from entering psychiatry [22]	**Item 18:** I would encourage a bright student to enter psychiatry.
If a student is interested in psychiatry as a career, other students or faculty will try to dissuade him or her [23].	
Psychiatry has a low prestige among the general public [22].	**Item 19:** Psychiatry has low prestige among other medical disciplines.
Psychiatry has high status among other medical disciplines [22].	
Psychiatry is a respected branch of medicine [21].	
At times it is hard to think of psychiatrists as equal to other doctors [21].	
Within medicine, psychiatry has high status [23].	
	**Item 20:** Many students at this medical school are interested in pursuing psychiatry as a career.
Many people who could not obtain a residency position in other specialties eventually enter psychiatry [22].	**Item 21:** Students who could not obtain a residence position in other specialties eventually end up in psychiatry.
On the whole, people taking up psychiatric training are running away from participation in real medicine [21].	
Psychiatrists tend to be at least as stable as the average doctor [21].	**Item 22:** Students are generally attracted to psychiatry because of their own personal problems.
Most nonpsychiatric faculty and house staff at my medical school are critical of psychiatry [23].	**Item 23:** My colleagues generally speak well of psychiatry.
Psychiatry is unappealing because it makes so little use of medical training [21,23].	**Item 24:** Entering psychiatry is a waste of medical education.
**Perceptions of psychiatric patients (n = 7)**	
Psychiatrists get less satisfaction from their work than other specialists [21].	**Item 25:** Working with psychiatric patients is rewarding.
I feel uncomfortable with mentally ill patients [22].	**Item 26:** Psychiatric patients are emotionally draining.
	**Item 27:** Psychiatric patients tend to be violent and unpredictable.
	**Item 28:** Psychiatric patients are highly appreciative of the care they receive.
	**Item 29:** Psychiatric patients should be treated in specialized facilities away from general hospitals.
Psychiatric patients are often more interesting to work with than other patients [21].	**Item30:** Psychiatric patients are often more interesting to work with than other patients.
Psychiatric illness deserves at least as much attention as physical illness [21].	**Item 31:** Psychiatric illnesses deserve at least as much attention as physical illnesses.
**Perceptions of Psychiatric Training (n = 6)**	
Teaching of psychiatry at my medical school is interesting and of good quality [22].	**Item 32:** Psychiatric teaching at this medical school is of the highest quality.
The majority of students report that their psychiatric undergraduate training has been valuable [21].	**Item 33:** Students at this medical school think that their psychiatric training has been valuable.
	**Item 34:** Less time should be spent in the medical curriculum teaching psychiatry to medical students.
	**Item 35:** Psychiatric rotations are well structured and informative.
Psychiatry is so amorphous that it cannot really be taught effectively [21].	**Item 36:** Psychiatry is so vague and imprecise it cannot really be taught effectively.
These days psychiatry is the most important part of the curriculum in medical schools [21].	**Item 37:** Psychiatric rotations should not be mandatory.

Following the scoring approach recommended by Ballon et al. to avoid non-committal response sets [22], items were rated on a 4-point Likert type agreement scale ranging from strongly agree to strongly disagree, with no neutral option. Several items were reversed to avoid response patterns. To minimize social desirability bias, respondents were asked what they thought others in their medical school would endorse. This approach, originally recommended by Link and Cullen [24], has been used extensively in population studies of stigma to minimize social responsibility biases that can emerge when asking people to make declarations of personal prejudices.

Items were translated and back translated by bilingual investigators in each setting. In some sites this was done by a single individual, and in others, by a small group. Two authors (HS and NS) independently reviewed the back translations for comparability to the original scale.

### Data management and analysis

Completed surveys were returned via email (scanned .pdf files) or by courier to Queen’s University, Canada, where they were entered and analyzed. Queen’s University Health Sciences and Affiliated Teaching Hospitals Research Ethics Board granted ethics clearance. In addition, some study sites also obtained local ethics reviews and clearances.

We first conducted an exploratory factor analysis. Because the results of exploratory factor analysis may not be replicable in a new sample (there is a tendency for models to over-fit the data), we randomly split the sample. Exploratory factor analysis was conducted on the first half of the sample and confirmatory factor analysis was conducted on the second half. Osborne and Fitzpatrick [25] refer to this as internal replication and recommend that researchers examine their exploratory factor analysis solutions using replication samples to determine the extent to which their solutions are likely to be robust. Items with strong loadings in the exploratory analysis may not load strongly in the confirmatory analysis and so may need to be dropped once the confirmatory factor analysis is completed.

Because the survey items were ordinal, we performed principal components factor analysis using the polychoric correlation matrix and varimax rotation. We examined eigenvalues (1.0 or greater), scree plots, and factor loadings to select two potential factors. Once the factors were selected we conducted two confirmatory factor analyses on the remaining half of the sample with structural equation modeling using Stata 13 [26] following the procedures described by Acock [27]. In the confirmatory factor analysis, we allowed for correlation between the factors as this occurs frequently even when varimax rotation has been used in the exploratory analysis to identify uncorrelated factors [28]. We examined a variety of goodness of fit statistics to assess the appropriateness of the model to the data. We eliminated items with poor factor correlations (under 0.4) and respecified the model based on model indices statistics. To obtain a better fitting model, we conducted a second confirmatory factor analysis allowing for correlated error variances between items within a scale whenever the modification indices were 10 or higher on the assumption that highly correlated items may also have correlated errors. We examined the wording of all correlated items to ensure that they made conceptual sense. We used Cronbach’s alpha to assess internal reliability of the resulting scales.

## Results

Table 2 describes the composition of the full sample. The majority of respondents were male (60.2%). Career stage was evenly distributed by design. Study sites were predominantly from Asia-Pacific and Eastern European countries, though a site in Scotland also provided data. In two instances (Russia and Thailand) more than one study site contributed data. We could determine the medical field for all but 32% (n = 339) of the sample, however the largest group (19%) was from an undisclosed specialty. Otherwise, the most frequently occurring fields were family medicine and surgery.

**Table 2:** **Sample composition**

**Table characteristic**	**Unweighted % (n)**
**Gender**	
Male	60.2% (482)
Female	39.8% (319)
Missing	258
**Career stage**	
Early	35.8% (329)
Middle	33.4% (307)
Late	30.8% (283)
Missing	140
**Site**	
Belarus	8.9% (95)
China	3.9% (41)
Croatia	15.0% (159)
India	2.5% (26)
Indonesia	3.5% (37)
Iran	16.0% (169)
Japan	2.8% (30)
Portugal	1.4% (15)
Romania	7.0% (74)
Russia	16.6% (176)
Scotland	3.2% (34)
Singapore	1.9% (20)
Thailand	12.1% (128)
Turkey	2.4% (25)
Ukraine	2.8% (30)
**Medical field**	
Anesthesiology	2.4% (17)
Chinese medicine	0.4% (3)
Community medicine	1.9% (14)
Dermatology	2.5% (18)
Emergency medicine	1.4% (10)
Family medicine	10.0% (69)
Lab	6.4% (46)
Neurology	4.3% (31)
Oncology	1.5% (11)
Pathology	4.3% (31)
Pediatric	8.8% (63)
Perinatal	6.1% (44)
Radiology	4.3% (31)
Rehabilitation	1.3% (9)
Unspecified Specialty	28.2% (203)
Surgery	16.3% (117)
Other	0.4% (3)
Missing	(339)

The Kaiser-Meyer-Olkin measure of sampling adequacy of .73 indicated that the survey items were sufficiently correlated to warrant conducting a factor analysis. Table 3 shows these results. Column 2 of Table 3 describes the factor loadings associated with the exploratory factor analysis, excluding any items with loadings less than 0.4. Two factors emerged with strong eigenvalues over 1.0 accounting for 54% of the overall variance. Factor one had an eigenvalue of 7.3 and factor 2 had an eigenvalue of 2.3. Three additional factors had eigenvalues exceeding 1.0 (1.8, 1.3, and 1.1 respectively); however the item loadings on these factors were sparse, conceptually inconsistent, and the alpha values were disappointing. Therefore, we chose a two-factor solution as the best fit for this dataset. Thirteen items were eliminated at this stage.

**Table 3:** **Factor loadings from the exploratory factor analysis (EFA), the confirmatory factor analysis (CFA) and the confirmatory model respecified (CFA2)**

**Factor**	**EFA** ^**1**^	**CFA** ^**2**^	**CFA2** ^**3**^
	**(N = 343)**	**(N = 415)**	**(N = 419)**
**Factor 1: Image of Psychiatry**			
1. Psychiatry is unscientific. (R)	.41	.72	.52
2. Psychiatry is not a genuine and valid branch of medicine. (R)	.51	.56	.60
3. Psychiatric treatments are not evidence-based. (R)	.47	.75	.64
4. There is very little psychiatrists can do for their patients. (R)	.57	.63	.53
5. Psychiatric hospitals are little more than prisons.	.48	.45	.52
6. Most psychiatrists are not clear, logical thinkers.	.60	.45	.58
7. Psychiatrists are not good role models for medical students.	.71	.73	.70
8. Psychiatrists are difficult to talk to.	.69	.48	.50
9. Psychiatrists are not attentive enough to physiology.	.71	**.28**	--
10. Psychiatry is filled with people whose medical skills are of low quality. (R)	.71	.60	.57
11. Psychiatry has low prestige among other medical disciplines. (R)	.51	**.37**	--
12. Students who could not obtain a residency position in other specialties eventually end up in psychiatry. (R)	.48	**.09**	--
13. Students are generally attracted to psychiatry because of their own personal problems. (R)	.47	**.34**	--
14. My colleagues generally speak well of psychiatry.	.41	**.39**	--
15. Entering psychiatry is a waste of medical education. (R)	.67	.76	.58
16. Psychiatry is so vague and imprecise it cannot really be taught effectively. (R)	.56	.63	.58
**Eigenvalue**	7.3		
**Alpha**	.83	.83	.82
**Factor 2: Efficacy of Psychiatry**			
1. Psychiatry is a rapidly expanding frontier of medicine.	.66	.49	.66
2. Psychiatric treatments are as effective as treatments in other branches of medicine.	.48	.47	.57
3. Most people who receive psychiatric treatment find it helpful.	.58	.57	.61
4. I would encourage a bright student to enter psychiatry.	.42	**.36**	--
5. Working with psychiatric patients is rewarding.	.52	.51	.54
6. Psychiatric patients are highly appreciative of the care they receive.	.51	.66	.42
7. Psychiatric teaching at this medical school is of the highest quality.	.48	**.19**	--
8. Students at this medical school think that their psychiatric training has been valuable.	.60	**.34**	--
**Eigenvalue**	2.3		
**Alpha**	.68		.61

^1^Rotated loadings (> .4) using varimax rotation. (R) = Reverse coding. Kaiser-Meyer-Olkin measure of sampling adequacy = .73.

^2^Chi square model vs saturated 1215, df = 448, p < .001; Root mean square error of approximation = .10; Comparative fit index = .54 Standardized root mean squared residual = .40; Coefficient of determination = .96.

^3^Chi square model vs saturated 216, df = 95, p < .001; Root mean square error of approximation = .055; Comparative fit index = .80 Standardized root mean squared residual = .13; Coefficient of determination = .95.

Items loading on the first factor were those reflecting negative stereotypic images of psychiatry, psychiatrists, and psychiatric residents such as psychiatry is unscientific; students are attracted to psychiatry because of their own problems; or entering psychiatry is a waste of a good education. We designated this scale the *Images of Psychiatry Scale*. Cronbach’s alpha for the items composing this 16-item scale showed high internal reliability in this sample (.83). The eight items that loaded on the second factor portrayed psychiatry as a rewarding and efficacious branch of medicine, such as psychiatry is a rapidly emerging frontier of medicine; most people who receive psychiatric treatment find it helpful; or working with psychiatric patients is rewarding. We designated this scale the *Efficacy of Psychiatry Scale* and noted it had acceptable reliability in this sample (alpha = .68). Because we used varimax rotation, the factors were uncorrelated.

Colum 3 of Table 3 summarizes the results of the first confirmatory factor analysis (CFA1) testing a model with two scales with uncorrelated error variances between items. Several of the key goodness of fit statistics indicated that the model was a poor fit for the data. The Chi-square statistic was significant (which often happens in samples over 200), the root mean square error of approximation (RMSEA) was well above the .05 threshold at .10, the comparative fit index of .54 was considerably less that the desired .90 threshold, the standardized root mean squared residual was greater than .05. In addition, 5 items on the Images scale and 3 items on the Efficacy scale had correlations of less than .40. This model explained 96% of the variation in the data.

Column 4 of Table 3 summarizes the results of the second confirmatory factor analysis (CFA2) using the same second half of the sample with a respecified model allowing for correlated errors within scales. The fit of this model was considerably improved. Though the Chi square statistics remained significant (as expected), the Root mean square error of approximation was reduced to .055, the comparative fit index rose to .80, and the standardized root mean squared residual dropped to .13. This model also explained 95% of the variation in the data. Internal consistency was high for the resulting 11-item Images Scale (.82) and acceptable but lower than desirable on the 5-item Efficacy Scale (.61), likely due to the smaller number of items. The final two scales were modestly correlated (.50).

**Figure 1:**
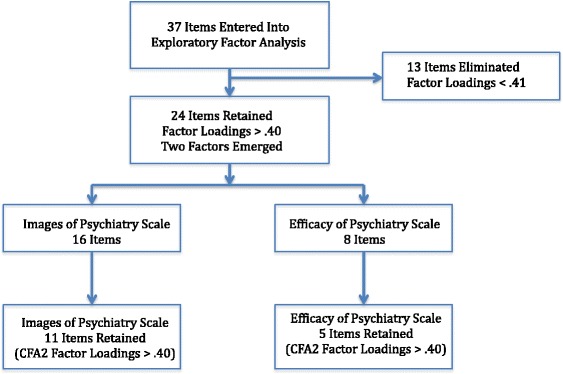
**Summary of exploratory and confirmatory factor analyses (Respecified model).**

Figure 1 summarizes the results of the exploratory and the confirmatory factor analyses in terms of the items retained at each step. It shows the results of the second and best fitting confirmatory factor analysis. Thirty-seven items were entered into the exploratory factor analysis. Thirteen were eliminated with factor loadings of .40 or less. The remaining 24 items loaded on two factors: 16 items on the Images of Psychiatry Scale, and 8 items on the Efficacy of Psychiatry Scale. On the basis of the second confirmatory analysis, 11 items were retained on the Images of Psychiatry Scale and 5 were retained on the Psychiatric Efficacy Scale. The specific items that were retained appear in Colum 4 of Table 3. Items that were eliminated appear in bold in Column 3 of Table 3.

## Discussion

There is growing concern over the shortage of psychiatrists worldwide and many organizations, such as the World Psychiatric Association, the American Psychiatric Association, and the European Psychiatric Association, have attempted to understand the reasons behind low recruitment levels among medical students [13]. For example, Farooq and colleagues recently studied the career plans of 2198 final year medical students in 46 medical schools from 40 countries. Across all countries, 4.5% of students definitely considered psychiatry as a career choice. Women, prior experience with a mental or physical illness, media portrayal of doctors, and positive attitudes to psychiatry were associated with a career choice of psychiatry. In order for the survey to be sufficiently brief to improve response rates, a number of factors, including potentially stigmatizing attitudes towards psychiatry and psychiatrists, were not addressed [10].

The scales developed in this research can be used to examine the attitudes of medical educators and their effects on psychiatry career choices of medical students, which is an important gap in our knowledge. Prior to this study, a psychometrically tested scale using both exploratory and confirmatory factor analysis did not exist. We identified two unidimensional scales. The first measures stereotypic *Images of Psychiatry* and psychiatrists. The second measures positive aspects of *Efficacy of Psychiatry*. The Images Scale was the longer and stronger of the two. As alpha values are influenced by the number of scale items, it is likely that the *Efficacy of Psychiatry Scale* could be improved in future research with the addition of a broader range of items.

A persistent question faced by those measuring attitudes has been the extent to which responses reflect socially correct responses, as opposed to the more deeply held attitudes that are embedded in our cultural views and more likely to govern behaviours. Link and Cullen have operationalized these deeper attitudes by asking respondents to indicate how they think “most people” would respond to someone with a mental illness. They found that, when asked directly, respondents tended to report idealized responses reflecting cultural norms for politically correct responses. When deeper attitudes were measured, scores were higher, reflecting greater stigma [24]. Based on this research, an important strength of our measurement approach was to ask medical educators to indicate the view that they thought best reflected the attitudes of their colleagues in their medical schools. Similarly, as recommended by Balon and colleagues [22] we deliberately excluded a neutral category in the agreement scale to avoid non-committal response sets.

A second strength of our approach was that we based our results on a large heterogeneous sample of medical educators drawn randomly from 23 academic settings in 15 countries. Survey items were translated and back translated following a systematic process with final approval from the principal investigators. This means we can have some confidence that these scales will behave well in a wide assortment of samples and educational settings. However, the test sites in this research did not include centres from countries such as Canada, the United States, or Western Europe, where the bulk of this research tends to occur. Also, there were a high number of missing values pertaining to socio-demographic characteristics and medical field, as these were not always obvious from the staff directories. Therefore, future research is needed to assess the usefulness of these instruments in a wider sample of North American and European countries.

## Conclusions

Using exploratory and confirmatory factor analyses, we identified two unidimensional scales to measure attitudes of medical educators toward psychiatry and psychiatrists: the *Images of Psychiatry Scale* (11 items) and the *Efficacy of Psychiatry Scale* (5 items). These constitute the first psychometrically tested scales to measure attitudes of medical educators—knowledge that is important if we are to better understand the determinants of low recruitment into psychiatry.
